# “*They don’t feel what I feel*”: lived experiences of women accessing comprehensive abortion care in pastoralist communities of Oromia region, Ethiopia: A Phenomenological study

**DOI:** 10.1371/journal.pone.0344436

**Published:** 2026-03-10

**Authors:** Tolasa Yadate, Abel Negussie, Yohannes Addisu Wondimagegene, Finina Abebe, Niguse Tadele, Yonas Abebe, Assefa Seme

**Affiliations:** 1 School of Public Health, College of Health Science and Medicine, Dilla University, Dilla, Ethiopia; 2 School of Public Health, Yirgalem Hospital Medical College, Yirgalem, Ethiopia; 3 Clinton Health Access Initiative (CHAI)-Ethiopian, Addis Ababa, Ethiopia; 4 School of Nursing, Addis Ababa University, Addis Ababa, Ethiopia; 5 Department of Midwifery, College of Health Science and Medicine, Dilla University, Dilla, Ethiopia; 6 School of Public Health, Addis Ababa University, Addis Ababa, Ethiopia; University of Warwick, UNITED KINGDOM OF GREAT BRITAIN AND NORTHERN IRELAND

## Abstract

**Background:**

Unsafe abortion is a significant global health concern, contributing to high rates of maternal mortality, particularly in developing countries. Women in pastoralist communities face unique challenges to access comprehensive abortion care (CAC) due to sociocultural, economic, and geographical barriers. This study explores the lived experiences of women in their journey to utilize CAC services in pastoralist communities of the Oromia region, Ethiopia.

**Methods:**

A phenomenological study was conducted in pastoralist communities of the Oromia region, including the Borana and Guji zones. A total of nine women were interviewed in-depth for their lived experiences in their journey to access the CAC service. All interviews were audio-recorded, transcribed verbatim, translated to English, and coded using Open Code version 4.03 software. Both inductive and deductive thematic analysis were employed to analyze the data. The lived experiences of women in their journey to access CAC services were analyzed under three categories: ‘before’, ‘during’, and ‘after’ utilizing the CAC service.

**Results:**

Before receiving CAC service, women faced emotional instability, fear, decision-making difficulties, and barriers such as transportation challenges, stigma, and lack of family support. During the service, women reported mixed experiences with healthcare providers, with some receiving supportive care while others reported disrespect and a lack of privacy. Following receiving CAC service, experiences included both relief and satisfaction for some, while others expressed regret and guilt due to cultural and religious beliefs surrounding abortion.

**Conclusion:**

The study reveals the complex experiences of women in their journey of accessing CAC services in pastoralist communities, worsened by socio-cultural norms, economic hardship, and limited healthcare infrastructure. Addressing the barriers identified, particularly those related to stigma, provider attitudes, and healthcare accessibility, is essential for improving CAC service delivery in these underserved regions.

## Introduction

According to the World Health Organization estimates, annually 42 million pregnancies end in induced abortion, from which 20 million end as unsafe abortions. Consequently, this results in 47,000 maternal deaths, 99% of which occur in the developing countries, and 5 million temporary or life-long disability [1]. Moreover, the users of comprehensive abortion care (CAC) can experience significant physical, social, and psycho-emotional challenges, which can be one of the major causes for unsafe abortion practices.

CAC refers to a range of medical services designed to ensure safe abortion procedures and post-abortion care, including counseling, contraception, and follow-up services [2]. It prevents unsafe abortions and reduces maternal mortality by providing patient-centered care. In Ethiopia, the revised abortion laws in 2005 under the Criminal Code of Ethiopia (Article 551) permit abortion services under specific circumstances, including cases of rape, incest, fetal abnormalities, or when the pregnancy poses a risk to the woman’s life or health [3,4]. Additionally, a woman can seek an abortion if she is a minor and unable to care for a child. Despite these legal bases for the service, women who access comprehensive abortion services experience different challenges in some areas due to stigma and healthcare resource constraints [3,5].

In many pastoralist communities, there is a strong social stigma surrounding abortion, which could lead to feelings of shame and isolation for women who seek abortion care [6]. Women may also face stereotypes from community members or healthcare providers. Additionally, pastoralist communities are located in remote or rural areas with limited access to healthcare facilities [7–9]. This can make it difficult for women to access CAC, leading to delays in seeking care or resorting to unsafe methods.

Women in general and pastoralist women in particular experience many difficulties before, during and after the CAC service, which have serious implications for their health as well as social life. In addition to the health problems, they may go through social judgments, physical challenges and health professionals’ ignorance at health facilities [10,11]. These challenges they experiences can have many consequences which has immediate or long-term impacts on their health and life, including deterioration in their health and wellbeing. Lack of social support and the community’s reaction toward their condition can also lead to a psychological crisis of the woman, which affects her social status in the community [12,13]. Furthermore, the lived experience of women who utilized the service can be illustrative of quality of CAC services [14,15].

However, this scenario is not well explained or demonstrated through qualitative research exploring the lived experiences of the women who undergo the condition. The lived experiences of women when accessing CAC in pastoralist communities would be complex, shaped by a range of social, cultural, and economic factors. Accordingly, healthcare providers and policymakers must take these factors into careful consideration when planning and delivering CAC services in these communities [7,16]. However, the evidence on this matter is insufficient for effective interventions. Effective responses to address those problems in pastoralist settings depend on understanding CAC users’ experiences to design evidence-based interventions. Therefore, this study aimed to explore the lived experiences of women who utilize comprehensive abortion care in the pastoralist communities.

## Materials and methods

### Study design and setting

Phenomenological qualitative research was conducted in the pastoralist or semi-pastoralist communities residing in the Oromia Region of Ethiopia. We used a phenomenological research approach to explore the lived experiences of women who accessed abortion. This study approach aims for depth and nuance rather than population estimates [17].

The total surface area of the pastoralist community accounts for 43.1% of the total surface area of the Oromia region. These communities include, but are not limited to, Borana and Guji [9]. The study included women from both rural areas as well as urban areas who accessed the CAC service.

### Study participants

The study participants for this study were women who accessed CAC services, both of whom had undergone unsafe and spontaneous abortion in pastoralist communities of the Oromia region. Those who had used the CAC service within the last month and one week before the data collection time were involved in the study. The maximum variation purposive sampling technique was applied to recruit the study participants to capture heterogeneity in key experience-shaping factors (residency and educational status).

In general, nine women who utilized the CAC service were interviewed to explore their lived experiences. A phenomenological study recommends (5–10 in-depth cases) to explore the lived experiences of the study participants [18]. We set an initial target of 10 participants, which is expected to yield rich information. However, by the 8^th^ interview, we observed codebook stabilization; no new codes were emerging. By the 9^th^ interview, we confirmed saturation, and so we stopped at n = 9.

### Data collection procedures

In-depth interviews were conducted from May 1 to July 10, 2024, using a topic guide. The topic guide questions were prepared by the authors and reviewed for contextual relevance. The women who visited public health facilities, both hospitals and health facilities, were interviewed in this study.

For data collection purposes, the heads of maternal and child health at health facilities were contacted and fully informed regarding the study so that they can fully inform women who utilized CAC services about the study during their visit for CAC services. These women were scheduled for an interview after one week to one month following their visit. Their contact information was recorded to remind them of their interview appointment. A reminder call was sent to them one day before their scheduled appointment. On the day of data collection, the women returned to the health facility for the interview. On the day of their appointment, the researchers conducted interviews to gather comprehensive information on their experiences in their journey to access CAC services.

### Data analysis

Verbatim transcription was done for audio recordings. Transcript files were read thoroughly and then coded by two independent coders. An initial code book was developed based on the reading and rereading of the transcripts. Coding was conducted using Open Code 4.03 software. Then, data was analyzed using inductive thematic analysis. Field notes that were taken during data collection were used to support the write-up of the findings. A thick description of the findings was carried out, and direct quotes from the interview data were presented to support the study findings. We have written the study findings categorizing under three categories: before, during and after receiving the CAC service. Under these categories various themes and codes of lived experiences of women were identified.

### Trustworthiness/rigor

The following rigor components were applied in this study to ensure the trustworthiness of the research process and findings. To ensure the credibility of the findings, training was given for the research assistants and the interview guide was reviewed and pre-tested to guarantee that the questions are contextually appropriate and relevant, reducing potential biases or misunderstandings. Field debriefings were being taken to help validate the data by allowing continuous reflection from different perspectives. To ensure dependability in data collection and analysis, an audit test of audio recordings and transcripts files and peer review were done to further strengthens the reliability of research process and findings. In terms of dependability, consistency was checked in the findings. A thick description of time, place, and context were reported in this research to allow the future researches to determine the transferability of the findings to different settings by understanding the study's contextual background.

### Ethical considerations

Ethical approval was obtained from the Institutional Review Board (IRB) of Dilla University to ensure that the research adhered to ethical guidelines (*Ref. No:* ***duchm/irb/001/2024***). Additionally, a support letter from the Oromia Regional State Health Bureau was secured, ensuring adherence to regional ethical standards and institutional backings. Considering the possible emotional impact of the study, participants were fully informed about the study's objectives, procedures, possible risks, and benefits before participation. They were allowed to ask questions and were assured that their participation was entirely voluntary, with the option to withdraw at any time without consequences. Written consent was obtained to document their agreement to participate.

Given the sensitivity of the research, ethical issues were a priority throughout the study, and several measures were taken to protect the rights, privacy, and well-being of participants. Strict confidentiality measures were implemented. Personal identifiers were anonymized to ensure that individual responses could not be traced back to participants. Data security was maintained through password-protected files and restricted access to authorized researchers only. Interviews were conducted in a private and secure setting to prevent any risk of social stigma or discomfort. The researcher was well-equipped to offer the appropriate emotional support to the participants if needed. Participants were reimbursed with 200 ETB to cover their transportation costs for their interview appointment. This financial compensation was assumed solely to cover expenses, rather than to serve as an undue incentive that could pressure individuals into taking part in the study.

## Result

A total of nine women were interviewed in-depth for their lived experiences when accessing CAC services. Those women who were interviewed include: two students, three self-employed, one government employee, and three housewives. The age range of those study participants is from 19 years old to 30 years old. Of the nine women, four participants had miscarriages, and five underwent unsafe abortions. The following table summarizes the findings under each category, along with themes and identified codes (Table 1).

**Table 1:** Summary of findings of lived experiences of women in their journey to utilize CAC services in pastoralist communities of Oromia region, Ethiopia, July 2024.

Categories	Themes	Codes	Note
Before service use	Transport related	Distance	Transportation issues like long distances, unavailability of transportation services, poor roads, and high costs are major problems.
		Transportation access	
		Transportation cost	
		Road facility	
	Psycho-social	Emotional instability	Emotional instability, fear, and limited support due to stigma and the secrecy of the issue.
		Fear	
		Social support	
	Awareness and decision making	Knowledge of legal issues	The lack of knowledge, secrecy of the abortion, and uncertainty lead to decision-making difficulties.
		Information seeking	
		Disclosure	
		Decision making	
During service use	Medical procedures	Rooms and spaces	High pain and discomfort, often in shared spaces, lack of privacy in most health facilities.
		Pain and discomfort	
		Privacy and dignity	
	Health providers	Healthcare providers’ interaction	Mixed experiences from healthcare providers, some supportive, others disrespectful.
		Healthcare providers’ attitude	
	Emotional state	Feelings	Stress and fear upon arrival at healthcare facilities.
After service use	Physical health	Slow recovery	Pain, bleeding, and slow recovery were reported.
	Psychological health	Emotional distress	Relief for some, guilt and regret for others due to cultural and religious beliefs.
		Relief and satisfaction	
		Guilt and regret	
	Social health	Support system	Social and relationship issues like stigma and lack of support from family and community were identified.
		Stigma	
		Community’s judgment	

### Category 1: Before service use

Most of the study participants reported that the journey from home to healthcare was not comfortable. Under this category, the following codes were identified: transport-related problems (distance, lack of transport services, transportation cost, and poor road facilities), uncertainty and emotional instability, fear, decision-making process, information seeking and disclosure, and social support and opposition (family, friends).

### Theme 1: Transportation-related issues

#### Distance.

The major barrier identified in this study was distance. According to the study participants’ response, they used to travel long journeys from home to the health facility to get CAC service. The study participant mentioned that they must travel a long distance to obtain this service. Most participants explained that their experience is related to the long distance they travel to access CAC services. Particularly, being in serious pain accessing the CAC service located at a very distant location from their residency was a major challenge.

*“…being in that difficult pain, bearing up the community’s judgments, it is challenging to travel a long distance to find a health facility” (Age 25, Housewife)*

*“…when they take me to the health center in our area, within about 20 kilometers, they said that we should go to another health center at the woreda uh…” (Age 28, Housewife)*

#### Transportation access.

Besides physical distance, participants mentioned problems related to transportation access. The study participants explained that the transportation facility was one factor that caused a barrier to CAC service use. They cannot use other options like walking to reach the health service areas because of the long distance, and their health condition does not permit it. Lack of transportation facilities caused them to stay at their home, experiencing critical pain and with huge bleeding, which led to a serious health condition.

*“… It is challenging to get transport services even for other purposes. You can find it maybe only on market days. Even when you are in good health, you cannot travel a long distance from home to find the health centers.” (Age 25, Housewife)*

*“… I stayed for three days at my home after seeing bleeding from my body. Because it is difficult to get transportation, you can find some motorbikes” (Age 28, Merchant)*

#### Transportation cost.

In addition, even after they secured access to a transport service, women further face a challenge in affording the cost of the transport. Most participants mentioned that they were requested to pay double when compared to other times. The reason behind this challenge was that since the women experienced serious pain, the car or motor drivers requested extra payment. The additional transportation fee was due to both the shortage of transport services and the woman's health condition.

*“…I paid double during my journey from home to health facility since I was almost unconscious and my blood continued to flow from my body*.” (*Age 30 housewife)*

*“…They cover my body with so many cloths, since there was a very high blood flow from my body, I paid 500 birrs for a motor, which was very expensive since it was very difficult to carry a woman whose blood flow was extremely high from the body.” (Age 28, Housewife)*

#### Road facility.

The other issue that the women faced during their journey was the hardship in relation to the road facilities. The road was poorly constructed, and there were ups and downs during their journey. As a result, the pain they experienced during the journey was very serious. They experienced high blood loss as a result of poor road infrastructure.

*“...On the way, I felt serious pain, stomach ache, since there was a high blood flow from my body, as I told you before, the blood was coming and stopping now and then, I felt just like a woman who had just given birth. The road was not comfortable.” (Age 25, Housewife)*

### Theme 2: Psycho-social support

#### Emotional instability.

Most of the study participants were in deep emotional stress when they became aware that their pregnancy was in trouble. Particularly those who experienced spontaneous abortion were emotionally unstable when they realized they were about to lose their baby. Many things come to their mind before they get the service, and they get confused and feel hopeless. The reason for this state of emotion was that the participants worried since they did not know what would happen to them even some study participants were crying when they were explaining their journeys to the health facility.

*“… No one was by my side, uh… (Crying) I didn’t tell anyone. I was not in my consciousness when I decided to travel to the health center for the service.” (Age 28, Merchant)*

*“…It was around five o’clock local time when I have seen blood flowing from my womb. Rather than the blood, I was concerned a lot about losing my pregnancy.” (Age 25, Housewife)*

#### Uncertainty.

Uncertainty regarding their health outcome after experiencing bleeding from their body was one of their major concerns before obtaining the CAC services. Their emotional distress is related to their uncertainty regarding their health outcomes after the service.

“…*when I was thinking to come to this health care facility, I was thinking whether or not I die, but in the moment, I arrived to this facility, what they did for me and what they speak to me was really good and relax me and I start to believe that my life does not pass away.” (Age 23, student)*

#### Fear.

Most of the women who want to have a CAC experience fear of visiting a health care facility. The fear emerges from various perspectives, such as the perception that they would get stigmatized by society and/or by medical procedure, and the potential pain they would experience during abortion. The women who experience abortion fear because they think that they may lose their lives. Moreover, they get depressed since the phenomenon was about discontinuing pregnancy and killing one's own baby. The condition was much worse by far among those who had undergone an induced abortion.

*“…I fear too much to come to the hospital. I fear the community’s judgment if they knew it. They devalue you and judge you according to their understanding. They don’t treat you as other women since then if they knew you experienced an abortion. And when you think about the things that health professionals do to you, uh… There are many things”* (*Age 30, Housewife)*

#### Social support.

Mostly for the married participants, their husbands were beside their wives before and during the abortion time, and they usually gave emotional, psychological, and economic support. The main reason behind this reality was mostly abortion case was kept secret between husband and wife. This is because the community perception towards any type of abortion was offensive and prone to judging the victim and relating the scenario of abortion with various factors, such as God's curse and the like. Unmarried women received social support mostly from one or two of their close friends.

*“…I even just feared telling anyone at the beginning, and finally, I told only my husband. I slept throughout the day, then the next day I was accompanied by my husband and went to the health facility.” (Age 25, housewife)*

*“…Usually, the community perceived that abortion was a very serious thing, and it may occur because of God's curse or punishment on that family. Everybody does not want to disclose it; as a result, I myself told only my two friends, and they were encouraging me.” (Age 23, student)*

### Theme 3: Awareness and decision making

#### Knowledge of legal issues.

The study participants who had an induced unsafe abortion mentioned that abortion was just equal to committing murder or killing a person. The knowledge and the attitude of respondent on the legal grounds of abortion seriously affected their experiences. Since abortion was not allowed unless they met the specified criteria by national legal basis, most of them used other traditional alternatives and self-attempt to end their pregnancy.

*“…I knew that if one person committed abortion, the legal body can bring that person to legal court, because it is not different from killing a human being.” (Age 23, Student)*

*“…Since it is illegal, I first received medicine from a pharmacy. The health center does not give the service. After the bleeding got worse, it was a must to go to the hospital.” (Age 19, student)*

#### Information Seeking.

Even though they want detailed information about the CAC service, the participants sought information and counseling from their husbands. In rare situations, particularly those unmarried, go to seek information and counseling from their very close friends. They seek health information regarding their condition when it gets worse.

*“…I discussed only with my husband after I had known that the case was becoming serious and I couldn’t withstand any more. After we reached to health center, the health providers told me my condition and I need some stay at the health center.” (Age 25, housewife)*

*“…My dorm mate told me I should go to the uh… hospital since I was becoming weak and couldn’t go to class.” (Age 19, student)*

#### Disclosure.

Most study participants took CAC secretly. Only they disclose their situation and uptake of the CAC service to their significant others, particularly to their husband, and very few friends. Those who practiced unsafe abortion didn’t disclose their decision to others. As a result, they tend to decide personally to use the locally available options to end their pregnancy. Since disclosure leads to various community judgments and stigma, they prefer to keep it secret.

*“…First, I used the medication bought from a pharmacy from a private clinic, and then, since the condition got severe, I went to the hospital. It was difficult, I only told to only one of my classmates since I am a student.” (Age 19, Student)*

*“…I haven’t shared the information for anyone, uh… because no one understands your problem deeply. When my neighbor asked me, I told them it was the increase in blood during my period. (The participant is crying).” (Age 28, merchant)*

#### Decision making.

The study participants were hesitating whether they should go to a health facility or not to get CAC service, and it was difficult for them to decide to terminate the pregnancy. It was hard to go to the health facility even though the service was available nearby their dwelling in rare scenarios. The decision of women who utilize CAC is influenced by personal, cultural, or religious beliefs.

Even though the country’s legal framework doesn’t allow them, they have different reasons to terminate their pregnancy, ranging from personal to socio-economic factors. The personal reasons include health problems they are experiencing, low income to raise a child, having already secured enough children, and an unplanned pregnancy. On the other hand, most participants mentioned that the societal culture and religion were the main factors that encouraged the women to deliver once the pregnancy had occurred in the woman's life. Since they cannot find abortion services from health facilities with those reasons, they use other traditional practices to end their pregnancy and then visit health facilities for CAC services when their health condition becomes complicated. On the contrary, those who experienced spontaneous abortion tend to be quicker to decide to travel to a health facility since their condition is unexpected.

*“…I decided this not due to my evilness, but I cannot help it rise. (The participant is crying). You know that child can lead even the country, if it were born.” (Age 28, merchant)*

*“…Since my culture and religion do not allow me to experience abortion, I was just fighting with myself whether I have to abort or not, but finally I decided to get an abortion to live and survive for the rest of my children.” (Age 30, self-employed)*

*“…The pregnancy was unplanned. Since my child was less than one year old, if I became pregnant with it, that would be a problem for the child. At the same time, I don’t have adequate money to raise them. I was hopeless when I found known I was pregnant. Uh… since I didn’t have any other option, I decided this.” (Age 25, merchant)*

## Category 2: Lived experience during service time

### Theme 1: Medical Procedure

#### Room and space.

Most of the study participants mentioned that the medical procedure was done in various situations; some said the procedure was done for them in an open space, and it was done in the same place where the normal delivery would be conducted. They also mentioned that it gives bad feelings to get the service in such settings. Room and space were a major concern for unmarried women than married women since they prefer a separate room to ensure their privacy due to the fear of the community’s judgment. In addition, the room for the service was not clean, and blood was scattered here and there inside the room.

*“…You know, it is difficult to abort while other mothers are delivering. It hurts your heart. They did the procedure for me at the open space where the laboring mothers deliver… I have got one person who knows me at the delivery ward, and I felt ashamed when she came. I tried to hide my face by covering it with my scarf.” (Age 19, student)*

*“…there is no more pain to be treated for abortion in the place where others deliver a baby. It hurts you so much. They don’t care; they treat wherever they want.” (Age 23, Student)*

#### Pain and Discomfort.

Regarding the pain and the discomfort, the study participant mentioned that they experienced very high pain, headache, and confusion since a huge bleeding came out of their body. Thus, the expression of the study participants in this regard was very serious. Most of the participants mentioned that they had never experienced such a difficult and painful condition in their entire lives.

*“…The level of red blood in my body was lower than the standard, the normal one was 12, mine was only 6, so they referred me to the hospital, then I received blood, I got the medical service that I deserve, but if I did not go to a health facility my life would be pass away.”* (*Age 25, Government employee)*

*“…It was very painful when they cleaned my abdomen (uterus), I lost my consciousness, and they gave me glucose. I have never seen such a painful situation in my life.” (Age 28, Merchant)*

#### Privacy and dignity.

Some of the study participants mentioned that their privacy and dignity were kept for them during the service. On the other hand, some participants mentioned that the privacy of the service was not maintained for them. Students and unmarried participants mentioned that their privacy was not maintained during the procedure.

*“…No one sees the process they serve me with big respect, the bedding and the room were very good, I did not face any problem yet, from this side. Everything was very good.” (Age 25, Government employee)*

*“…I was somewhat comfortable when I was at the emergency room, but when I came to the delivery ward, it created another impression. I have got one person who knows me at the delivery ward, and I felt ashamed when she came. I tried to hide my face by covering it with my scarf. You know it is so hard, because once the information was disseminated within the community, you cannot be treated similarly.” (Age 19, student)*

### Theme 2: Healthcare providers’ approach

#### Care.

Regarding the CAC service, most of the study participants appreciate the service they have received from health care providers. Some participants complained that the health professionals offended them; on the other hand, some participants just praised the health professionals and admired the service they received from the health facility. They mentioned that if they did not come to the health care facility, a huge amount of blood would get out of their body, in addition, they would be exposed to a very serious health problem. On the other hand, some others complain that the service they received was not complete because the health care facility lacks the necessary items.

*“…With God’s help, it is those health professionals who saved me from death. My condition was so serious since I came after staying three days at my home. Only some of them ignore you. There is no listening machine (ultrasound); they only cleaned my uterus.” (Age, 28, Merchant)*

*“…I took all the services, but the problem is what I face during my stay in the hospital, the healthcare providers don’t care about your feelings. Their words break your heart.” (Age 30, Self-employed)*

#### Attitude.

The healthcare providers’ attitude during the CAC was not uniform; some of the participants mentioned that the healthcare providers really supported them. Others mentioned that they didn’t treat them well. They mentioned that healthcare professionals were not respectful in their approach, and they did not give attention, and they were ignorant. Moreover, they mentioned that they did not get information about the service from health professionals.

The participants stated that they become hurt psychologically by the negative words and disrespectful approaches of health professionals. The participants were in deep emotion when they were explaining their experiences with health professionals. They mentioned that the psychological pain is more hurting than the physical pain. Those who had undergone the self-induced abortion reported experiencing disrespectful treatment from healthcare providers. The participants explained that they will not come back to that hospital, even for other health services.

*“…They just support me with good intentions and a happy face, if they did not show me a good face even though I did not tell them what was inside my heart, rather I just hold inside my heart and I fear them since they are health professionals and respected personnel. But their reaction was very nice to me.” (Age 25, Government employee)*

*“…Around the emergency room, when I reached there, one nurse insulted me, saying, “Why did you do this and stay this long time?” She overlooked me. They said, “Go out from here, this is not the place to get such service.” I have been there since I was in a very critical condition. I was thinking if I could get the right person who treats me. But they were not interested in helping me. I was saying, “Why do they ignore me, why don’t they care for me?” Finally, I understood that they ignored me due to my case since it is an evil act. So, I decided to tolerate it since then****.”*** (*Age 28, merchant)*

*“…To relieve my pain, I received another pain uh… I don’t think I will come back to this hospital, because the way they hurt your psychology with their words and ignorance is difficult. That is more painful than the pain you came for treatment. Uh…instead, they were blaming me while they were giving the service. (The participant is crying).” (Age 19, student)*

### Theme 3: Emotional state

#### Feelings.

The study respondent mentioned that they get stressed when they arrive at the gate of the health facility for CAC service. Some even cried since they were disturbed emotionally. The reason behind this emotional disturbance and crying was that they feared something might happen to their life, even though they suspected that their life might pass away. They mentioned that they will not come back to the health facility for the same case. Generally, after abortion service, the reaction of the women was not the same. They mentioned that they were not feeling well while they were using CAC services.

*“…I was stressed when I was in the compound. When I was taken to the place where the pregnant and laboring women are treated, I was not feeling comfortable, because I am not the mother who is going to give birth.” (Age 19, student)*

*“…I was crying when they were asking me why I had done this. I was in shock. So, they gave me a blood transfusion, and then I recovered from the problem.” (Age 28, merchant)*

*“…health professionals really upset me. So, it is very painful for me. At that moment, I blamed myself being experiencing abortion has its own negative effect on your mind, but when the words of others add to this, it is really difficult to imagine what you feel. Uh…anyways, it is difficult to explain (Oh!)” (Age 19, Student)*

## Category 3: After receiving the CAC service

### Theme 1: Physical Health

#### Slow recovery.

The study participants mentioned that they really felt pain after CAC and even though they had been exposed to back pain. Whereas some women mentioned that they recovered fully from their condition. Moreover, the study participants mentioned that they had experienced various health problems like severe headache, anemia, abdominal cramps, and high blood loss. They have been recovering after they received the necessary health services and medication.

*“…I really feel pain after abortion***;** *I did not get out of bed for at least 15 days. The bleeding and the pain were so intense. I feel back pain until now.” (Age 30, Self-employed)*

*“…I am in good health after the abortion service. But I am not recovering completely because I feel some pain like headache, stomachache, and the like.” (Age 25, Government employee)*

### Theme 2: Psychological Health

#### Relief and satisfaction.

The study respondent mentioned that they got relief and felt good after the abortion service, because they escaped from the worry and depression of raising the child. The two main reasons they primarily mentioned that caused them to get an abortion, specifically for a self-induced abortion, were economic hardship to support their child, and the occurrence of an unplanned pregnancy. So, they mentioned that to some extent, they get relief from their stress and worry about the child that is going to be born. Satisfaction of unmarried women and those who attend their education resulted from being able to be saved from the stigma of the community.

*“…I am satisfied with the service I received, and I am so happy regarding this scenario. Overall, since I decided by myself to end the pregnancy, I was very happy. And I decided not to come to this facility with the same case since I was regret on what I did.” (Age 30, Self-employed)*

*“…As I told you before, even when I came here, I came with two ideas. I had taken the drug from the pharmacy before I came to this hospital. I feared that “do they [hospital] accept me or not?” but it is beyond my expectation. The good thing was I stopped the baby from coming to this world, because he gets relief from stress. Thus, I am really satisfied with the service I have received.” (Age 25, Merchant)*

*“…If I continued the pregnancy, I could not continue my education. Above that, I would be disrespected among the community. That has many impacts on my entire life and future life.” (Age 23, student)*

#### Guilt or regret.

The feeling of guilt and regret after CAC was very common since the religion and morality of the participants did not support this service, particularly the induced abortion. Most of the study participants believed that they had an abortion because they didn’t have any other option at all. No one encourages using abortion services, and they promise themselves not to repeat the abortion.

*“…I internally blame myself, but I would not do such acts again in my life. I know that conducting an abortion is like killing of human being. That is why I feel guilty. Really, I am guilty. But I can do nothing but this. (She is crying).” (Age 19, Student)*

*“…I was feeling guilty about this act when I remembered the Sharia laws, since it didn’t recommend such acts. So, I feel that God will punish for this. But I can do nothing, since I am poor and cannot raise my children, so I should do this. The older child itself is not capable of getting on its feet.” (Age 25, Housewife)*

### Theme 3: Social Health

#### Stigma.

Stigma after use of the CAC service was common. The stigma may come from the family members or from the surrounding community. Since the women fear stigma, they did it in secret. The participants explained that their family members stigmatized them after they used CAC services. The community members were not advised to contact those who had an abortion, due to the belief that they can share those evil experiences.

*“…My older sister was upset when she heard that I had an abortion, even though she stopped coming to my home, and she did not pick up my phone still.” (Age 30, Self-employed)*

*“…Both elders and religious fathers may tell the other community members not to interact with her. Because they believe that the women can share that bad experience with the community. So, this may cause one to be isolated from the community.” (Age 27, Merchant)*

#### Community judgment.

No one encourages conducting an abortion in society. The legal framework of the country on this matter is also restrictive. However, women prefer to conduct an abortion using other traditional practices, believing that they do not have any other option in their lives. Even the victim did not believe in abortion. The community perceives terminating pregnancy as an evil act. They talk behind a person who has experienced an abortion. The community believes that a child is a gift of God, and advises that the pregnancy should not be discontinued. The community recommends delivering the baby even if the pregnant woman is poor and does not have money to raise the child. Similarly, the religion advises women not to abort. The religious leaders teach that abortion is a sin in front of God’s/Allah’s eye, and they blame the person who experiences abortion. This community’s belief about abortion has a significant impact on the experience of women who use CAC services.

*“…Rather, they advise raising children once they are born, and they do not allow you to end pregnancy at all because, as you know, religion says just “reproduce and become too many” so it does not allow, it does not permit abortion. Sometimes, the community believes that if the women abort spontaneously, they perceive that it is a curse on her. They perceive that God is paying back for her bad works.” (Age 23, Student)*

…*From the religious perspective, I feel really sorry and I regret after I did an abortion. The religion does not encourage abortion at all; it is a very serious thing from the religious perspective*. (*Age 25, Merchant*)

## Discussion

This study examined the lived experiences of women during their journey to access CAC services in pastoralist communities of the Oromia Regional State, Ethiopia. Accordingly, the result was classified into three major categories: the experience of women before they arrived at the healthcare facility, the experience during service use at the health facility, and the experience of study participants after using the CAC service.

Transportation-related issues were the major challenge women experienced in their journey to utilize CAC services. While the previous studies stated the distance and transport challenges as major problems in pastoralist communities while accessing health services [7–9], our findings suggest that these challenges are much worse in the case of CAC service users. Unlike other routine health services, the CAC service is less widely available, requiring women to travel longer distances, often while experiencing huge bleeding or emotional distress. So, they should travel to another health facility that is at a longer distance than the one available to them relatively nearby. Again, on top of that, the transportation cost is a major problem related to transport challenges. They should use rental services to reach the facility where they get services. This implies that the unavailability of the CAC services at their nearby locations exacerbates women’s vulnerability and delays care. These findings reinforce the justification from global literature that geographic inaccessibility disproportionately affects the disadvantaged rural and pastoralist women [2].

Beyond structural barriers, women’s narratives revealed that they were exposed to uncertainty, fear, and emotional instability. The fear and uncertainty were caused not only by the anticipated pain of medical procedures but also by the anticipated stigma and moral judgment. Prior research from South Africa and other low- and middle-income countries has identified abortion stigma as a central determinant of women’s psychological burden [16,19]. Our findings confirm this evidence but extend it by showing that stigma is about total isolation in pastoralist settings, where the community structures magnify women’s fear of disclosure. This underscores the importance of introducing stigma reduction and prevention approaches to CAC in Ethiopia, going beyond individual counseling to broader community-level interventions.

Information seeking and disclosure about abortion further reflected the interrelation of stigma and marital status. They mostly obtain the information from the most intimate person; married women often relied on their spouse for information, while unmarried women turned to their peers. This reliance on intimate social networks rather than formal health information mediums shows a gap in accessible, stigma-free information sources on reproductive health, particularly the legal restriction on abortion. Further, this implies that the level of their fear of stigma and community judgment in this case. This is not merely an individual shortcoming but points to systemic deficiencies in health communication, particularly around family planning and abortion. This is related to broader debates on gender equity: when women cannot independently access reliable information, their autonomy in reproductive decision-making remains constrained. Previous studies underscored similar evidence regarding the disclosure and information seeking of CAC service users [20,21].

The study also highlights limited knowledge about the legal grounds of abortion in Ethiopia. Although Ethiopia’s legal framework regarding abortion has been revised, allowing abortion only under the specified conditions, participants’ lack of knowledge reflects inadequate dissemination and community-level engagement. Similar gaps have been reported in other East African countries [22], suggesting that legal reform alone is not adequate without consequent community sensitization. In our study, the gap contributed to reliance on unsafe abortion practices and created the perception of abortion as morally and religiously impermissible. This finding adds to the existing literature by showing how legal restrictions, cultural norms, and weak health system communication interplay to continue to encourage unsafe abortion practices. This result was supported by the studies with a study from South Africa and other contexts [16,23].

Women who had planned to have an induced abortion reported difficulty in deciding to seek CAC services, largely because abortion is neither culturally nor religiously supported in their communities. This is consistent with other studies, where moral, cultural, and religious norms strongly shaped their decision-making moral compass [16,24]. Married women often decided to seek abortion due to economic hardship, while unmarried women were motivated by fear of community judgments, stigma, and discontinuation of schooling. Socioeconomic constraints were a common influence on decision-making, echoing findings of other studies [25,26].

Social support also varied by marital status. Married women tended to receive support from their spouse, and usually kept it secret due to community disapproval of abortion, which was often associated with a curse from the divine. On the other hand, unmarried women are limited to their close friends rather than family, fearing negative responses and social labeling, which is consistent with prior studies [16,25]. This highlights the impact of marital status in shaping abortion experiences. The absence of supportive social networks increases women’s vulnerability to isolation and unsafe practices. These findings suggest that strengthening confidential counseling, peer support, and community-level stigma reduction could mitigate the social burden of abortion, particularly for unmarried women.

Women’s accounts of providers’ care and attitude were mixed, ranging from appreciation of support to reports of ignorance and lack of privacy. This inconsistency with healthcare providers’ approach is in line with findings from Ethiopia and elsewhere that provider attitudes are shaped by their personal beliefs and inadequate training [27,28]. Our study extends to the literature that the lack of separate spaces for CAC within facilities, forcing women to receive care in delivery rooms, contributes to perceptions of compromised dignity and privacy. The implication is not only infrastructural but ethical: healthcare institutions demand independent abortion facilities that would foster a more encouraging atmosphere for both patients and healthcare professionals [23,29–35].

Finally, the post-abortion experiences of women ranging from relief and satisfaction to guilt and regret show the dual emotional trajectory of abortion in stigmatized settings. This is consistent with other studies that describe abortion as “both a liberating and distressing experience” [16,25,36]. However, in our study, lack of social support triggered feelings of guilt and isolation, specifically for unmarried women, because it imposes the belief that the woman is disgusting and promiscuous [23,37]. This points to the importance of broader community engagement to familiarize post-abortion support and to promote gender equity in reproductive healthcare rights and services uptake.

### Limitations of the study

The scope of this study covers the lived experiences of women with CAC services, from before they received the services to after they completed them, which could make it lengthy and difficult to manage.

Even though the interview was conducted in a private and secure setting, scheduling participants at the health facilities where they received services might limit their ability to fully share the experiences they had while accessing CAC services.

## Conclusion

The lived experiences of women in their journey to accessing CAC service were identified to be characterized by various conditions, including, before arriving at a health care facility: distance, lack of transportation services, transportation cost, poor road facility, emotional instability, fear, difficulty to decision making, information seeking, disclosure, and social support and opposition from family and friends. During service use, experiences such as pain and discomfort from medical procedures and environments, negative health care providers’ interaction and attitude, and lack of privacy and dignity. Finally, after using the CAC service, different experiences like slow health recovery, emotional and psychological consequences, and social and relationship-related problems.

The lived experiences of women in their journey to access CAC services were characterized by various conditions, including before reaching healthcare facilities: distance, lack of transportation, transportation costs, poor road infrastructure, emotional instability, fear, difficulty in decision-making, seeking information, disclosure, social support, and opposition from family and friends. During service use, experiences included pain and discomfort from medical procedures and environments, negative interactions and attitudes from healthcare providers, and a lack of privacy and dignity. Finally, after using the CAC services, women faced different experiences such as slow health recovery, emotional and psychological consequences, and social and relationship-related problems.

The infrastructure of CAC services should be developed (i.e., a dedicated room should be prepared for CAC services from the delivery ward to protect the privacy and confidentiality of service users). Capacity building should be given to healthcare professionals on the CAC services and the patient approach when they treat CAC cases. Community health education and awareness raising should be provided for the community on the Ethiopian legal framework of abortion services. Further studies should be conducted on the implementation and impacts of Ethiopia’s current legal frameworks on abortion, particularly its association with the practice of unsafe abortion.

## Supporting information

S1 File Supporting Information File. (RAR)
